# Proceedings of Endocrine Surgical Course held at JPMC Karachi Pakistan from June 17-19^th^ 2013

**DOI:** 10.12669/pjms.294.4017

**Published:** 2013

**Authors:** Shaukat Ali Jawaid

Karachi: Jinnah Postgraduate Medical Centre Karachi organized a three day Endocrine Surgical Course from June 17-19^th^ 2013. Prof. Woong Youn Chung, Professor of Surgery and Director of Thyroid Cancer Clinic at Yonsei University College of Medicine, Seoul Korea was the invited guest speaker and Master Trainer. This 3-Day interactive Course with live demonstration of various surgical techniques in different endocrine disease attracted a large number of general as well as ENT surgeons from all over the country including junior as well as senior surgeons besides postgraduates. It provided them a rare opportunity to learn about different surgical procedures to manage thyroid from the visiting Korean Master Trainer Prof. Chung. 

**Clinical Features and Management of Benign Thyroid Goiter**

Prof. Woong Youn Chung in his presentation on clinical features and management of benign thyroid goiter disclosed that during the Year 2012 they at their center managed 2649 cases of thyroid which included 2504 malignant and 145 benign thyroid. The surgical management includes conventional thyroidectomy and endoscopic thyroidectomy. There were a few cases of parathyroid as well. Goiter is defined as enlargement of thyroid gland which was first reported in China in 2700 BC. Main cause is iodine deficiency. He then discussed various classification of Goiter based on WHO system and said that almost 75% of all swellings are simple goiter. If the prevalence of goiter is more than 5% in any area, it is known as endemic area for goiter. In developing countries iodine deficiency is the main cause. Daily requirement of iodine is between 0.1 to 0.15mg. Use of iodized salt has helped to overcome this deficiency of iodine to a great extent.

For management of simple goiter, no treatment is required and one must keep these patients under observation. Iodine supplementation of food, vegetable, milk is recommended. The treatment is to suppress the TSH and reduce gland volume by 50%. Goiter regrowth occurs rapidly after therapy cessation. Radioactive iodine is useful in 30-80% of patients. Treatment consists of thyroxine PEI/RFA which are some of the non-surgical managements of benign thyroid goiter. Surgical treatment is the last choice when the nodules have cosmetic problem and then there are symptom pressure nodules with radiation exposure.

Hyper thyroidism or toxic goiter is predominantly a disorder in women. He also discussed its clinical features in detail. Grave’s Disease is diffuse toxic goiter which is seen mostly in young patients between the ages of 20-40 years. Some of causative factors are genetics, smoking, pregnancy and infection etc. Toxic multinodular goiter is also known as Plummer’s disease. He then also referred to toxic adenoma. For diagnosis and management, thyroid function testing is essential. There is low TSH; T4 T3 has to be increased. In medical therapy radio iodine treatment is useful. There are various treatment modalities medical as well as surgical and each one of it has some benefits and risks involved. Treatment is with beta blockers, anti thyroid drugs. Pregnancy and breast feeding were some of the absolute contra indications for use of radioactive iodine.

Talking about surgical therapy, Prof. Chung said it is indicated in case of large obstructive goiter, in cases with failure after medical therapy, those who are not fit for Radioactive Iodine treatment like pregnant women. Anti thyroid drugs have to be taken for about six weeks. Lugol iodine is taken for two weeks before operation. In surgery, sub-total thyroidectomy is one of the options. Prog.Chung then talked about hypothyroidism and Thyroditis in detail and said that it was an infection like viruses and bacteria. These patients present with weight gain, constipation, edema and cold intolerance. Some of the symptoms also include weakness and dry skin etc. Hashimoto’s Thyroiditis was first described in 1912. It is a chronic autoimmune disease. It is painless firm diffuse goiter. Silent Thyroiditis may be confused with post partum Grave’s disease. In sub acute Thyroiditis oral steroids, NSAIDs and beta blockers are quite useful as regards symptoms of hyper thyroidism. Acute supportive Thyroiditis is 70% bacterial. These patients have severe neck pain, fever and focal thyroid tenderness. Riedel’s Thyroiditis is also painless goiter seen in middle age. Treatment is resection for compressive symptoms along with use of tamoxifen and other drugs. Treatment of benign thyroid conditions is improvement of their symptoms, low morbidity and improvement in their quality of life. One should always aim at optimal management of benign thyroid goiter, he remarked.

**Advanced Thyroid Surgery**

On June 18^th^ second day of the course Prof. Wong Chung first made a presentation on advanced thyroid surgery and discussed in detail Endoscopic Thyroidectomy and Robotic Thyroidectomy. Talking about changes and evolution of surgery he said that first it was open surgery which was followed by minimally invasive surgery and then came the era of computer aided or Robotic surgery. Conventional open thyroidectomy has been very safe. Then the surgeons started doing minimally invasive open thyroidectomy (MIOT) ET is also safe and feasible but it has some limitations. He then discussed the cervical approach and other approaches for endoscopic thyroidectomy which were developed by various Japanese and Korean Surgeons. Gasless axiliary approach, he pointed out is excellent as regards cosmetic effects and gives natural view as open surgery. During November 2001 to December 2011, they did 1085 cases at their center as cosmoses intended surgery. Talking about limitations of endoscopic thyroidectomy, Prof.Ghung said that it is a more difficult procedure, there are limitations of space for work, and it is more invasive hence chances of complications are more. There is also difficulty in complete thyroidectomy and securing the LN dissection. 

As regards benefits of robotic surgery, the operator can control all the procedures. Preservation of parathyroid gland is possible. He then explained his own surgical technique which he has developed with some modification and he was using it since October 2007. He also presented a comparison of endoscopic vs. robotic thyroidectomy as both the procedures have their benefits as well as limitations. Dissection has to be very careful in both the systems. Further advances in surgical techniques resulted in single incision procedure. From 2007 to 2013 so far they have done 3409 cases which included one hundred and four benign cases while all others were malignant. He further stated that now the number of cases of robotic surgery being done all over the world is increasing and mostly they are general surgery cases. Robotic thyroidectomy as a new single modality, he opined, is feasible and can be performed safely but the cost is very high in both the groups i.e. endoscopic as well as with robotic thyroidectomy. Robotic thyroidectomy is now feasible and safe to do and till 2011 we have done over seven thousand cases, he added. The limitations of robotic thyroidectomy include high cost, it is difficult to learn, it is more invasive. Now new instruments have been developed for robotic surgery and the surgeons need extensive training. He concluded his presentation by stating that they have started an international training programme for robotic thyroidectomy and soon they will be moving to a new purpose built building.

Replying to a question Dr. Wong Chung remarked that he uses 45 degree endoscope in Korea. Robotic thyroidectomy is more advanced as compared to endoscopic thyroidectomy. Responding to another question he said that selection of the patients for various procedures has to be very careful and he stages his patients by CT scan before taking a decision as to which procedure will be performed on a particular patient. Later two video films of Robotic thyroidectomy and endoscopic thyroidectomy were shown to the participants before he started showing live surgical procedures.

**Liver Surgical Demonstration**

Prof. Woong Youn Chung who had come with his team which included Dr. Son and Ms.Lee Technician also demonstrated liver different surgical procedures on June 17th, 18th and 19th. These included simple lobectomy, block neck dissection total thyroidectomy, endoscopic lobectomy, parathyroid adenoma and Cistrunk operation for thyroglossal cyst. During the discussion it was stated that large size tumours are difficult to dissect through endoscope. Responding to a question Prof. Chung said that he does both malignant as well as benign cases through endoscopic procedures with this approach of going through the axilla. Some of the cases which he operated were quite difficult but he had excellent results with clean dry surgery. His handling of the soft tissues with respect was remarkable. 

**Prof. Sikandar’s Technique**

**Prof. Sikandar Ali Sheikh** a noted surgeon from Larkana also made a brief presentation on how he manages thyroid and the technique which he has developed. It was supplemented with a Video presentation. He pointed out that so far he had operated upon two thousand thyroids. He does it as an open procedure as against the suture less surgery which Prof.Chung had demonstrated which is much more advanced. His technique, Prof. Sikandar Sheikh said was indicated for the unilateral benign goiter in the middle age or in elderly where scar is not a problem. It is also indicated for ablation of remnant of thyroid tissue in malignant goiter where lobectomy has already been performed. In the elderly patients post operative scar is hardly visible and this technique is highly cost effective, he remarked.

At the end of the course a number of participants expressed their appreciation for organizing this academic activity at JPMC. **Prof.Riaz Husain Dab **from Faisalabad said that they learnt many new surgical procedures and surgeons working at all the academic institutions should be aware of this. We may not be doing all these but some of the units which have endoscopes and other instruments can make an attempt and try to do this in carefully selected cases. Students can also be shown these surgical procedures live in the lecture rooms direct from the operation theatre which is not possible to show to more people in the operation theatre. He opined that tertiary care institutions at Lahore and Faisalabad should try to follow this practice started by JPMC at Karachi.

**Dr. Anjum **from Indus Hospital Karachi remarked that the young surgeons are lucky as they have seen these instruments like Hormonal scalpel in their early stages of their career while this facility was not available to us while we were in training. Juniors should learn it as robotic surgery is going to be the way surgery is performed in the days to come. A delegate from Peshawar said that they had learnt many new things during the course.

**Dr.Shaukat Malik** from Baharia Medical and Dental College said that he was not used to this endoscopic thyroidectomy but the way Prof.Chung had demonstrated it is important that we must keep the nerve in sight and this will minimize the chances of any complications. I intend to buy hormonal knife for myself. His other advice to the juniors was to ligate the vessels which go to the thyroid during surgery.

**Prof. Sikandar Sheikh** from Larkana said that we have witnessed Korean Transfer of Technology to Pakistan through the courtesy of Prof. Chung and his team. We need to increase the scope of such academic activities and through video conferencing these surgical procedures can be shown live all over the country so that many people can benefit from it. **Dr. Iftikhar** from Nishtar Medical College Hospital Multan said that by witnessing these live surgical procedures, their horizon has widened. We would have loved to see more cases being operated provided time management was better. Most of the junior surgeons would have preferred more open cases, he remarked.

**Prof.Shamim Qureshi** said that since Prof.Chung had developed his own technique of endoscopic thyroidectomy, he wanted to demonstrate it and many senior surgeons were also keen to see that. That is why we had more endoscopic cases. He introduced some endoscopic technique for the audience many of whom had seen it for the first time. Some of the cases were very difficult but he managed it exceptionally well providing the participants a rare opportunity to observe and learn all this. **Dr.Rafique** from Patel Hospital also appreciated the efforts of the organizers and said that juniors who cannot get hormonal scalpel should not be depressed but they should use other options.

**Prof. Muzzafar** from Lady Reading Hospital Peshawar said that it has provided us an opportunity to share our knowledge and experience. I always bring my unit staff and junior doctors to these meetings so that they can learn all this because in the days to come they are going to manage the patients and provide us the services. He requested all other senior surgeons coming from different cities to bring with them junior colleagues and even get them sponsored so that they can learn all this.

**Prof. Tasnim Ahsan** Director of JPMC in her comments said that she believes in perfection and meticulous execution. While managing some cases I had to look for surgical guidelines as to what is the best procedure for different cases. I feel that once I refer a case to surgical colleagues, I should not have to bother and let the surgical colleagues manage it the best way. Before attending this course, had I seen some patient with scar in the axilla and asked him/her which surgery they had and if they had said thyroidectomy, I would not have believed it but it is a reality. These are all new things and techniques and we are grateful to Prof.Chung and his team to have demonstrated all this lives to such a large audience.

**Prof. Zafar Ali Chaudhry** from Sialkot said that new technologies and surgical procedures will eventually come to Pakistan and we all should be aware of this. He commended the cases presented in the Consultant Corner which were very well worked up and it shows that JPMC has the best and perhaps the only endocrinology center in Pakistan. Now with the addition of endocrine surgery facilities, this is going to become a Center of Excellence in Endocrinology. **Prof. Omar** from Lahore said that looking at the majestic state of the art auditorium and the facilities available at the Emergency Operation Theatre is wonderful and this is perhaps the best Emergency Operation Theatre in Pakistan. 

 Prof. Shameem Qureshi and Dr. Naseem Baloch thanked all the Operation Theatre staff, technicians, anaesthesia team and doctors in the Ward who worked hard to make the programme a success who were also presented certificates of appreciation. They also thanked the representatives of the Pharmaceutical industry who had extended their valuable help and assistance in this course and in recognition of their services, they were also presented shields. In fact many of the participants enjoyed and learnt a lot from open procedures performed by Prof. Prof. Woong Youn Chung as they could immediately adopt some of the techniques they saw and start practicing. Even one of the participants remarked that they would have loved to see more open procedures rather than endoscopic thyroidectomy and robotic surgery as they require lot of initial investment and instrumentation which only few tertiary care institutions can afford but it is nice to see and witness these advances in surgery, they remarked.

**Concluding Session**

Prof. Wong Chung, Dr. Son and Ms.Lee were presented mementoes in the concluding session. Speaking at this occasion Pro.Wong Chung thanked for the kind hospitality extended to his team during their stay in Pakistan. I am particularly very impressed that even senior surgeons are so enthusiastic to learn to improve their surgical skills, he remarked.

**Inaugural Session**

Earlier speaking in the inaugural session on June 17^th^ 2013, Prof. Tasnim Ahsan Director JPMC pointed out that complete diagnostic and treatment facilities for various endocrine diseases was now available at Jinnah Postgraduate Medical Center Karachi. Prof. Tasnim Ahsan thanked Prof. Chung and his team for visiting Pakistan to impart training and share their knowledge and experience with colleagues in Pakistan. She then remarked that it was in 1992 that she first started the endocrine clinic at JPMC and now it has developed so much that it has been approved for postgraduate training in endocrinology. Doctors working at various endocrine units in different city hospitals have been meeting regularly where interesting cases are discussed and we have been working in close collaboration. At the same time, our surgical colleagues have been working at their own in this area and we need to increase our collaboration between different endocrine units at various city hospitals for such an academic activity. Now entire range of diagnostic work up and treatment facilities are available here at this center, she added. In the field of endocrinology we are seeing very exciting times. Organizing such an academic activity is not easy and that too when there is a very little support from the institution as our resources have been stretched to its limits.

**Prof. Mumtaz Maher** an eminent surgeon who in fact initiated such an academic activity many years ago at JPMC by inviting eminent colorectal surgeons to train younger people and also show live surgical procedures was the next speaker. He pointed out that demonstrating surgical procedures live is quite challenging. It provides an opportunity to the participants to see what is happening in the operation theatre. Surgery has to be seen to learn. Prof. Chung is a leader in thyroid surgery and an excellent operator. He will demonstrate his skills of surgery. Thyroid surgery, he further sated, was now being done by ENT surgeons, General Surgeons but many were not yet aware of the existence of parathyroid. 

**Dr.Shameem Qureshi** in his introductory remarks referred to endocrine surgery which late Prof.Sami Ashraf used to do at JPMC. Later Prof. Mumtaz Maher introduced, developed and expanded facilities for colorectal surgery at JPMC and we were able to invite six eminent colorectal surgeons from various countries who showed us their surgical skills while performing various colorectal surgical procedures which were all shown live to the participants from the operation theatre. He thanked the pharmaceutical trade and industry for their support which has helped them to organize this academic activity for which there is no registration fee for the participants. **Dr. Naseem Baloch** said that during the last year they examined eighteen hundred patients of which almost 85% were of thyroid diseases.

